# Ovarian reserve after uterine artery embolization in women with morbidly adherent placenta: A cohort study

**DOI:** 10.1371/journal.pone.0208139

**Published:** 2018-11-29

**Authors:** Aya Mohr—Sasson, Maya Spira, Rony Rahav, Dafna Manela, Eyal Schiff, Shali Mazaki-Tovi, Raoul Orvieto, Eyal Sivan

**Affiliations:** 1 Department of Obstetrics and Gynecology, Sheba Medical Center, Tel-Hashomer, Israel; 2 Sackler School of Medicine, Tel-Aviv University, Tel-Aviv, Israel; University of Washington, UNITED STATES

## Abstract

**Objective:**

To evaluate ovarian reserve in women after preservative cesarean delivery using uterine artery embolization due to morbidly adherent placenta.

**Study design:**

A historical cohort study including all women admitted to a single tertiary care center, with morbidly adherent placenta that had preservative cesarean delivery with bilateral uterine artery embolization. Inclusion criteria included gestational age >24 weeks, singleton pregnancy and placenta increta / percreta. Exclusion criteria included maternal age > 43 years old and cesarean hysterectomy. Control group included women attending the infertility clinic due to male factor or single women conceiving via sperm donation, matched by age. Blood samples were collected on day 2–5 of menstruations for hormonal profile and Anti Mullarian Hormone (AMH) levels. Primary outcome was ovarian reserve evaluated by the levels of AMH.

**Results:**

59 women underwent preservative cesarean delivery using uterine artery embolization during the study period. 21 women met inclusion criteria (33.9%) and were matched controls (n = 40). Circulating levels of E2 and FSH did not differ significantly between the two groups (p = 0.665, p = 0.396, respectively). AMH was lower in the study group (median 0.8 IQR 0.44–1.80) compared to the controls (median 2.08 IQR 1.68–3.71) (p = 0.001). This finding was consistent in linear multivariate regression analysis where the group of cesarean delivery using bilateral artery embolization due to placenta accrete was significantly predictive for the levels of AMH (B = -1.308, p = 0.012).

**Conclusion:**

Women post preservative cesarean delivery using uterine artery embolization due to placenta accrete have lower ovarian reserve compare to controls matched by age.

## Introduction

Placenta accreta is an abnormal adherence of the placenta to the underlying myometrium [1]. It is an increasingly prevalent and potentially dangerous complication of pregnancy associated with adverse maternal outcome, including life-threatening maternal hemorrhage, massive blood transfusion, uterine rupture and peripartum hysterectomy [2–4].

The management strategy for placenta accreta is a challenging problem in obstetrical practice. Prenatal diagnosis of abnormal placentation and a planned cesarean delivery have been proven to improve maternal outcome [5, 6]. Despite the use of effective therapies and procedures to control hemorrhage at cesarean delivery, planned hysterectomy is considered by many physicians as the gold standard procedure to treat women with morbidly adherent placenta [7–9].

A conservative management of morbidly adherent placenta can be considered when fertility preservation is desired [1]. Different techniques have been introduced, including uterine artery embolization (UAE) using radiographic identification of the bleeding vessels and occluding their flow with gelfoam, coils, or glue [10]. Prophylactic, intraoperative UAE before placental expulsion appears to reduce the risk of postpartum hemorrhage, decreases morbidity and mortality, and increases the chance preserving the uterus in patients with morbidly adherent placenta [11, 12].

Concerns have been raised regarding the effect of the embolization on ovarian tissue due to collateral vessels connecting the uterine to the ovarian arteries and possible flow of sclerosants to the connecting blood vessels. Data estimating the ovarian reserve after successful conservative treatment for morbidly adherent placenta are limited.

To the best of our knowledge, no prospective studies have specifically evaluated ovarian reserve following uterine artery embolization during cesarean delivery due to morbidly adherent placenta. The aim of this study is to evaluate ovarian reserve in this population.

## Materials and methods

The study protocol was approved by the "Sheba Medical Center" Institutional Review Board (ID 3178-16-SMC) on the 30 of July 2017, and was supported by the National Institutes of Health (NCT02821702).

This is a historical cohort study including all pregnant women admitted to a single tertiary care center, between November 2011 to July 2016 with morbidly adherent placenta that had preservative cesarean delivery with bilateral uterine artery embolization that was defined successful due to succeeding in uterus preservation. Inclusion criteria included: 1. Gestational age >24 weeks; 2. Singleton pregnancy; 3. Elective cesarean delivery with prophylactic pelvic artery catheterization of internal iliac arteries through femoral approach; 4. Renewal of menstruation after operation or a minimum of six months since operation. Exclusion criteria included: 1. Maternal age ≥ 43 years old at the time of recruitment for the study; 2. Hysterectomy due to the procedure. Women were identified based on medical records. All women in the study group were diagnosed with morbidly adherent placenta by sonographic evaluation, and in case of uncertainty, magnetic resonance imaging was added for estimating the final invasiveness through the myometrium. All operations were done electively. Invasive radiology team catheterized through femoral approach the internal iliac arteries, reaching the uterine arteries bilaterally before cesarean delivery was started. After delivery of the fetus, embolization was done in order to control hemorrhage. Control group was assembled from women attending the IVF clinic for infertility treatment due to male factor or healthy single women requiring sperm donation, matched by age to the study group. Women in this group was recruited after assembling of the study group in order to achieve age matching. The decision to use this control group was based on the assumption that this group was expected to reflect the general population fertility status, having no fertility problems, however, needing to complete fertility workup due to life circumstances before receiving treatment.

Enrollment for the study group took place in the obstetrics' clinics after giving telephone consent to participate in the study. Controls were enrolled during their visit at the IVF clinics. Blood samples from both groups were collected on day 2–5 of menstruation for hormonal profile and Anti Mullarian Hormone (AMH). Clinical characteristics and blood samples were compared between study and control group.

Concentrations of Estardiol (E2), Progesterone (P), Follicle Stimulating Hormone (FSH), Lutenizing Hormone (LH) and AMH were determined in a single accredited Mega Lab. Normal blood level at early follicular phase for E2, P, FSH, LH were defined as 73–308 (pmol/l), 0.1–3.6 (nmol/l), 3–14.4 (IU/l), 1.1–11.6 (IU/l) respectively. AMH was measured using Beckman Gen II ELISA kit with normal range values of 0.3–10.8 (micq/l). Primary outcome was defined as the ovarian reserve evaluated by the levels of the FSH and the AMH.

### Statistical analysis

Normality of the data was tested using the Shapiro-Wilk or Kolmogorov-Smirnov tests. Data are presented as median and inter-quartile range (IQR). Comparison between unrelated variables was conducted with Student's t-test or Mann–Whitney U test, as appropriate. The chi-square and Fisher's exact tests were used for comparison between categorical variables. Linear multivariate regression analysis was used to determine which factors were significantly and independently predictors with the level of AMH. Significance was accepted at p < 0.05. Power was calculated post hoc given α of 0.05. Statistical analyses were conducted using the IBM Statistical Package for the Social Sciences (IBM SPSS v.19; IBM Corporation Inc, Armonk, NY, USA).

## Results

During the study period 59 women underwent preservative cesarean delivery using uterine artery embolization due to morbidly adherent placenta. Seven patients underwent cesarean hysterectomy, twenty were 43 or older at the time of recruitment for the study, nine patients were lost to follow up and three refused to participate in the study. Ultimately, the study group included 21 women that met the inclusion criteria. None of the women in the study group needed fertility treatment before operation. 16 were operated due to placenta percreta, and 5 for placenta increta. Median estimated amount of blood loss during operation was 2000 mL (500 to 9000 mL). No major catheterization-related complications were reported in the study group. Median time for post operation blood sampling was 55 months (IQR 22.5–90.5). One woman was breastfeeding, 2 had intra uterine device (one of them hormone releasing) and all other women reported they were not using hormonal treatment for contraception at the time of blood sampling.

Table 1 displays the demographic and clinical characteristics of the women in the study group compare to the controls. The study group had higher gravidity and higher rates of cesarean delivery as expected (2 IQR:2–4.75 vs 1 IQR:0–1, p = 0.001), (1IQR:1–2 VS 0, p = 0.001), respectively. FSH levels were comparable between study group (5.8 IQR: 4.45–9.55) and control (5.59 IQR: 4.40–7.30, p = 0.396). AMH was found significantly decreased in the study group (0.80 IQR: 0.44–1.80 vs. 2.08 IQR: 1.68–3.71, p = 0.001). Two women in each of the groups had AMH values below normal.

**Table 1:** Demographic and clinical characteristics.

	Morbidly adherent placenta (n = 21) (IQR)	Control (n = 40) (IQR)	P
Age (years)	32 (30–36)	31(29–37)	0.670
BMI (Kg/m^2^)	22.49 (20.59–25.40)	23.1(20.30–26.60)	0.748
Gravidity	2(2–4.75)	1(0–1)	0.001
Parity	1(0–2)	0(0–1)	0.156
Previous CD	1(1–2)	0(0–0)	0.001
FSH	5.8(4.45–9.55)	5.59 (4.40–7.30)	0.396
LH	3.4(2.85–5.50)	4.2(2.80–5.90)	0.823
AMH	0.80 (0.44–1.80)	2.08 (1.68–3.71)	0.001

Data are presented as median and interquartile range (IQR).

BMI—Body Mass Index, E2- Estradiol, FSH- Follicle Stimulating Hormone, LH-Luteinizing hormone, AMH- Anti Mullerian Hormone, CD- Cesarean Delivery

The association between AMH and possible predicting factors was further evaluated using a linear regression model (R^2^ = 0.277, F (3, 55) = 7.01, P<0.001). Consistent with previous analysis, and although largely explained by age, cesarean delivery using uterine artery embolization due to placenta accreta was found to be an independent variable highly predictive of AMH levels (p = 0.008) (Table 2). Previous cesarean delivery was not included as an independent variable, because no previous cesarean deliveries have been reported in the control group. Power for the difference in AMH levels calculated post hoc based on the mean AMH levels 1.44(SD±0.31) for the study group and 2.92(SD±0.35) for the control group, reached 99% using sample size of 21 and 40 women for the morbidly adherent placenta and the control group, respectively.

**Table 2:** Linear regression analysis.

	B	95%CI		p
		LOWER	UPPER	
Age	-0.152	-0.258	-0.045	0.006
UAE* intervention	-1.308	-2.319	-0.297	0.012
FSH	-0.151	-0.299	-0.004	0.045

Dependent Variable: AMH

*UAE- Uterine Artery Embolization

## Discussion

The prevalence of morbidly adherent placenta has increased steadily during the past several decades, most likely secondary to the rising rate of cesarean deliveries–and currently occurs at a rate of 1:500 deliveries [13]. Our study group included women with high grade of placental invasiveness–increta, in which placental villi extend into the myometrium, and percreta, where the villi penetrate through the myometrium to the uterine serosa and may invade adjacent organs, such as the bladder [14]. Abnormal placentation is correlated to worse adverse outcome, the most disturbing is severe obstetric hemorrhage owing to the incomplete placental separation, which usually necessitates hysterectomy [15,16]. Prenatal diagnosis and adequate planning, particularly in high-risk populations, is indicated for the reduction of adverse outcomes [14, 17,18].

Cesarean hysterectomy is considered the most common planned treatment for morbidly adherent placenta, however, in young women who want the option of future pregnancy, conservative treatment is a valid option [19]. Different techniques have been used, including temporal internal iliac occlusion balloon catheters, post-operative methotrexate and prophylactic embolization of the internal iliac or the uterine arteries [10, 12, 20]. Prophylactic pelvic artery catheterization and embolization in women with morbidly adherent placenta was found to be safe and effective in prevention of hysterectomy and reducing massive hemorrhage [20]. Due to this findings, the use of conservative treatment with UAE for patients having placenta accreta and interested in preserving fertility, has become more common.

Sentilhes et al conducted a retrospective multicenter study on 93 women who had conservative treatment for morbidly adherent placenta [21]. 8.3% of women had severe intrauterine synechiae and were amenorrheic. Only 27 (28.12%) wanted future pregnancies, of them 24 had had 34 pregnancies, with a mean time to conception of 17.3 months (range, 2–48 months).

Not much have been studied on pregnancy success rate specifically after embolization in pregnancies complicated with morbidly adherent placenta. From studies on conservative treatment of uterine fibroids, it used to be a consensus, that recommendations concerning the treatment of women intending to have children, expressly reject the use of UAE [22]. Some recommendations view the wish to have children as a relative contraindication for UAE [23] or recommend pregnancy, only if the patient is closely been followed [24]. Recently, reports of successful pregnancies after UAE for conservative treatment of uterine fibroids have been published due to the growing experience [25, 26].

One possible concern for the potential risk of UAE to fertility is the exposure of the ovaries to ionizing radiation, being a procedure that is carried out under fluoroscopic control using contrast media for X-ray angiograms S1 Video. The radiation exposure of the ovaries is around 60–80 mGy [27]. The radiation dose during UAE depends very much on the experience of the radiologist [28]. Tse and Spies reviewed current studies, experimental investigations and comparative calculations of radiation exposure during UAE and came to the conclusion, that standard UAE procedures do not damage the ovaries [29].

Other mechanism potentially harming ovarian function using UAE may be explained by the unintentional migration through anastomoses of embolization material between the uterine and ovarian vessels, resulting reduced blood flow to the ovaries [27]. This is of great concern due to the fact that pregnancy amplifies the blood supply to the uterus [30]. According to review of the literature by Payne et al, temporary amenorrhea or complete ovarian failure after UAE treating uterine fibroids occurs in 1–14 % of cases [31]. A recent compilation of 15 studies published between 2004 and 2008 showed an age-dependent rate for permanent amenorrhea of 0–40 % [23]. In most cases amenorrhea was caused by cessation of ovarian function. This serious side-effect mainly occurred in women older than 45 years with reduced ovarian reserve which made them more susceptible to an embolic insult [32]. Nonetheless, Tulandi and Salamah reported an incidence of premature menopause due to impaired ovarian function after UAE of 1–2 % in women younger than 45 [33].

To the best of our knowledge this is the first study that evaluates hormonal status after UAE in patients with morbidly adherent placenta. Tropeano et al. presented 2 prospective studies on the long-term effect of myoma embolization on patient hormonal status. The first included 36 patients with long-term follow-up (60 months), that found no significant differences in ovarian size and FSH or E2 levels in women between the ages of 25 and 39 after embolization compared to a control group [34]. The second study included cohort of 43 patients with follow-up of 7 years, did not find evidence that fibroid embolization advance the timing of menopause in women before the age of 45 years [35]. AMH, a potentially more important parameter, was not evaluated in both of the studies [36]. These findings are in agreement with our results showing no significant difference in E2 or FSH in univariate analysis, suggesting no damage to ovarian reserve. However, in contrary to our findings, in multivariable logistic regression analysis, FSH and UAE were found to be independent predictors for the AMH levels, demonstrating a reduction in ovarian reserve.

Anti-müllerian hormone is considered the best endocrine marker for assessing the age-related decline of the ovarian pool in healthy women although its predictive value for future live births remains controversial [36–38]. Iwase et al describes AMH as a reliable marker of ovarian reserve, which may be useful for the assessment of ovarian toxicity due to medical and surgical treatments [39]. It is a stable marker, mostly not influenced from demographic, lifestyle, and menstrual cycle phase [40].

Lately, a few studies have been published specifically measuring the AMH levels after UAE for symptomatic uterine fibroids. Kim et al. found that younger ovaries (according to biological ovarian age) exhibit a greater capacity for recovery after ovarian damage in 3 month compared to 12 month follow up after UAE [41]. Similar results were observed by Mclucas et al. that conducted an observational study of 89 women, 23–40 years of age who received UAE. He found no significant change in AMH levels measured prior and after the procedure (mean time for measurement 190±229 days), and concluded that it does not affect ovarian reserve in women <40 years old [42]. Tsikouras et al. conducted a prospective case control study on 120 premenopausal women aged 40–50 years, who underwent UAE for symptomatic uterine fibroids that were compared to 120 matched controls. No statistically significant decrease was noted in AMH values 12 months post procedure, and no statistical significant alterations was observed in AMH values between the two groups [43]. In contrary to these studies, estimating ovarian reserve after UAE for fibroid treatment, we found AMH to be reduced in the UAE group. The difference might be explained by differences in technique, in the quantity of gel foam used or ionized radiation that the ovaries were exposed to. Furthermore, the median follow up since operation was much longer in our study (55 month).

As expected, age was highly predictive to the AMH levels in regression analysis. Although this strong relation and in addition to it, our study still found a specific effect of UAE on ovarian reserve that was statistically significant. The superiority of AMH in estimating ovarian reserve, can explain why it was found significantly reduced in the study group compared to the controls, while significance was not reached in the FSH levels.

The study has several limitations. The study group was relatively small due to the rarity of the condition. Patients were not allocated randomly for treatment, thus, results might have been biased by patients' treatment selection. And finally, the time for blood sampling since the operation was not uniform to all women in the study group.

The strength of this study is in it being the first to evaluate women's ovarian reserve after cesarean delivery due to morbidly adherent placenta using bilateral uterine arteries embolization. This study is also the first to include evaluation of AMH levels after embolization procedure for any gynecological intervention. Furthermore, the study has a control group that is double the size of the study group and consist women without intrinsic fertility problem that are matched in the most important factor affecting fertility–the age.

## Conclusions

The results of this study indicate that UAE in morbidly adherent placenta might may be associated with reduced ovarian reserve. Nevertheless, the alternative of hysterectomy is definite, and do not offer solution to women desiring future fertility. Our findings support the use of UAE in this patients, while giving the proper consultancy on the possible effect of reduced ovarian reserve. This information should be of value to physicians and patients alike. Further investigation need to be done to strengthen this finding.

## Supporting information

S1 Video Uterine artery embolization procedure. (MP4)

S1 File Protocol. (DOCX)

S2 File TREND statement checklist. (PDF)

S3 File Dataset. (XLSX)
