# Supplementary material for: Ovarian reserve after uterine artery embolization in women with morbidly adherent placenta: A cohort study
Source: PLoS One. 2018 Nov 29;13(11):e0208139. doi: 10.1371/journal.pone.0208139 (PMC6264507; doi:10.1371/journal.pone.0208139)
Supplement: S1 File — (DOCX) [file pone.0208139.s002.docx]

Study 3178-16-SMC, Protocol - Number #3, 1/7/2016

Fertility post preservative cesarean section and uterine artery embolization procedure in women with placenta accrete

Introduction:

Placenta accreta is an abnormal adherence of the placenta to the uterine wall. Three variants of abnormally invasive placentation have been described: placenta accreta, in which placental villi invade the surface of the myometrium; placenta increta, in which placental villi extend into the myometrium; and placenta percreta, where the villi penetrate through the myometrium to the uterine serosa and may invade adjacent organs, such as the bladder[^1^](#_ENREF_1).

Placenta accrete is an increasingly prevalent and potentially dangerous complication of pregnancy. It appears to be most strongly predicted by a history of cesarean deliveries and low-lying placenta/previa^[2](#_ENREF_2)^. In a large retrospective study including 34,869 CD, of which 0.4% were complicated with placenta accrete, prior cesarean deliveries and placenta previa were found independent risk factors for placenta accreta^[3](#_ENREF_3)^. Additional risk factors include in vitro fertilization pregnancy, prior myomectomy, Asherman's syndrome, submucous leiomyomata, maternal age older than 35 years, elevated second-trimester levels of α-fetoprotein and β-human chorionic[^2^](#_ENREF_2)^,^[^4^](#_ENREF_4)

Pregnancies complicated with placenta accrete are associated with adverse maternal outcomes, including life-threatening maternal haemorrhage, large-volume blood transfusion, uterine rupture and peripartum hysterectomy[^5^](#_ENREF_5)^,^[^6^](#_ENREF_6). Moreover , strong association was found between abnormal placentation to significant perinatal morbidity and mortality such as small for gestational age, preterm delivery, neonatal intensive care unit hospitalization, perinatal death and neonatal death[^7^](#_ENREF_7).

Prenatal diagnosis and adequate planning, particularly in high-risk populations, is indicated for the reduction of these adverse outcomes. Advances in grayscale and Doppler ultrasound have facilitated prenatal diagnosis[^8^](#_ENREF_8). Despite advances in imaging techniques, no diagnostic technique affords the clinician complete assurance of the presence or absence of placenta accreta.

A population-based descriptive study using the UK Obstetric Surveillance System (UKOSS) conducted a cohort of 134 women that were identified with placenta accreta, increta, or perccreta: 50% (66/133) were suspected sonographicly to have this condition antenatally. In women with a final diagnosis of placenta increta or percreta, antenatal diagnosis was associated with reduced levels of hemorrhage (median estimated blood loss 2750 versus 6100 ml, P = 0.008) and a reduced need for blood transfusion (59 versus 94%, P = 0.014), possibly because they were more likely to have preventative therapies for hemorrhage and were less likely to have an attempt made to remove their placenta[^1^](#_ENREF_1).

Management of placenta accrete could be conservative (aiming for uterine preservation) or interventional (elective cesarean hysterectomy) depending on the patients will to maintain the uterus for future fertility, the degree of placentation abnormality or complications during delivery. The extent (area, depth) of the abnormal attachment will determine the response—curettage, wedge resection, medical management, or hysterectomy. Uterine conserving options may work in small focal accretas, but abdominal hysterectomy usually is the most definitive treatment.

Post-partum hemorrhage is the major concern dealing with placenta accrete. Women with placenta accreta have a higher incidence of postpartum hemorrhage and are more likely to undergo emergency hysterectomy [^9^](#_ENREF_9) .

If the diagnosis or a strong suspicion is formed before delivery, a number of measures should be taken including counseling the patient about the likelihood of hysterectomy and blood transfusion, preparing blood products and clotting factors and considering using cell saver technology. The appropriate location and timing for delivery should be considered to allow access to adequate surgical personnel and equipment and a preoperative anesthesia assessment should be obtained[^10^](#_ENREF_10).

A patient with stable vital signs and persistent bleeding, especially if the rate of loss is not excessive, may be a candidate for arterial embolization. Radiographic identification of bleeding vessels allows embolization with Gelfoam, coils, or glue. Balloon occlusion is also a technique used in such circumstances. Embolization can be used for bleeding that continues after hysterectomy or can be used as an alternative to hysterectomy to preserve fertility.

Studies estimating the fertility and pregnancy outcomes after successful conservative treatment for placenta accrete have demonstrated placenta accreta does not appear to compromise the patients' subsequent fertility or obstetrical outcome[^11^](#_ENREF_11).

Doumouchtsis et al[^12^](#_ENREF_12), conducted a systematic review evaluating menstrual and fertility outcomes following the surgical management of postpartum haemorrhage. They included 28 studies: 17(n=675) after uterine artery embolization, 5 (n=195) after uterine devascularisation, 6 (n=125 ) following uterine compression sutures. 91.25% women resumed menstruation within 6 months of delivery. 77.87% women who desired another pregnancy achieved conception. They concluded that uterine-sparing radiological and surgical techniques for the management of severe PPH do not appear to adversely affect the menstrual and fertility outcomes in most women; however, the number of studies and the quality of the available evidence is of concern.

By reviewing the literature, no prospective studies have specifically evaluated fertility, following uterine artery embolization during cesarean section due to placenta accrete .

Our study is the first to investigate the fertility outcome and the influence on ovarian reserve after this operative procedure using uterine artery embolization during cesarean delivery.

Study design:

Prospective case control study

Inclusion Criteria:

Study group - Women with diagnosis of placenta accrete after successful cesarean section with bilateral uterine arteries embolization.

Control group - women attending the IVF clinic for infertility treatment due to male factor, or single requiring sperm donation, matched in age to the study group.

Exclusion criteria:

- Age- >43
- Hysterectomy due to the procedure

Methods:

Women that were operated in our center and fit inclusion criteria will be invited to participate in the study after getting notice in advance by letter on requirements. After approving interest in participating on telephone invitation, they will be invited to the monitor unit in the obstetric department scheduled on day 2-5 of their menstrual period. On their visit they will meet one of the research team that will explain the rational of the study and study requirements. After giving signed informed consent to participate in the study, demographic parameters and medical history will be taken, including - age, BMI, parity, gravity, past medical history, past operations.

Preoperative intra operative and post-operative information will be collected, including:

Pre -op – placenta location, degree of accrete, estimation of fetal weight, amniotic fluid index

Intra-op – Duration of operation, accrete degree, estimation of blood loss, Number of trials of embolization, total amount of material embolized, length of imaging (exposure to radiation)

Post op – Duration of hospitalization, complication (fever, hemorrhage, infection rate, other)

Late post op – month from operation, menses (regularity, amount of bleeding, duration of bleeding, metrorhagia, menometrorahgia ), pregnancy, spontaneous abortion, second operation,( delivery) .

All women participating will complete –

1. Day 2 Hormonal profile - FSH, LH, E2, Progesterone
2. Antral follicle count
3. AMH

Women from the control group will be recruited by one of the IVF physicians that are part of the research team during their visit at the IVF unit. As part of the conventional inquiry before starting treatment - blood samples and vaginal ultrasound will be taken as mentioned above.

Primary outcome:

Pregnancy rate after cesarean section with bilateral uterine artery embolization compared to control group

Secondary outcomes:

Parameters of ovarian reserve in the study group compared to control group

Reference:

1. Fitzpatrick KE, Sellers S, Spark P, Kurinczuk JJ, Brocklehurst P, Knight M. The management and outcomes of placenta accreta, increta, and percreta in the UK: a population-based descriptive study. *BJOG.* Jan 2014;121(1):62-70; discussion 70-61.

2. Eshkoli T, Weintraub AY, Sergienko R, Sheiner E. Placenta accreta: risk factors, perinatal outcomes, and consequences for subsequent births. *Am J Obstet Gynecol.* Mar 2013;208(3):219.e211-217.

3. Balayla J, Bondarenko HD. Placenta accreta and the risk of adverse maternal and neonatal outcomes. *J Perinat Med.* Mar 2013;41(2):141-149.

4. Clark SL, Koonings PP, Phelan JP. Placenta previa/accreta and prior cesarean section. *Obstet Gynecol.* Jul 1985;66(1):89-92.

5. Bauer ST, Bonanno C. Abnormal placentation. *Semin Perinatol.* Apr 2009;33(2):88-96.

6. Oyelese Y, Smulian JC. Placenta previa, placenta accreta, and vasa previa. *Obstet Gynecol.* Apr 2006;107(4):927-941.

7. Vahanian SA, Lavery JA, Ananth CV, Vintzileos A. Placental implantation abnormalities and risk of preterm delivery: a systematic review and metaanalysis. *Am J Obstet Gynecol.* Oct 2015;213(4 Suppl):S78-90.

8. Rao KP, Belogolovkin V, Yankowitz J, Spinnato JA. Abnormal placentation: evidence-based diagnosis and management of placenta previa, placenta accreta, and vasa previa. *Obstet Gynecol Surv.* Aug 2012;67(8):503-519.

9. Zaki ZM, Bahar AM, Ali ME, Albar HA, Gerais MA. Risk factors and morbidity in patients with placenta previa accreta compared to placenta previa non-accreta. *Acta Obstet Gynecol Scand.* Apr 1998;77(4):391-394.

10. Gynecologists ACoOa. ACOG Practice Bulletin: Clinical Management Guidelines for Obstetrician-Gynecologists Number 76, October 2006: postpartum hemorrhage. *Obstet Gynecol.* Oct 2006;108(4):1039-1047.

11. Sentilhes L, Kayem G, Ambroselli C, et al. Fertility and pregnancy outcomes following conservative treatment for placenta accreta. *Hum Reprod.* Nov 2010;25(11):2803-2810.

12. Doumouchtsis SK, Nikolopoulos K, Talaulikar V, Krishna A, Arulkumaran S. Menstrual and fertility outcomes following the surgical management of postpartum haemorrhage: a systematic review. *BJOG.* Mar 2014;121(4):382-388.
